# Pharmacological Potential of Peruvian *Eustephia* Species (Amaryllidaceae): Alkaloid Diversity, Cholinesterase Inhibition, and Anti-*Trypanosoma cruzi* Activity

**DOI:** 10.3390/plants14223510

**Published:** 2025-11-18

**Authors:** Olimpia Llalla-Cordova, Javier E. Ortiz, Mauricio Piñeiro, Luciana R. Tallini, Laura Torras-Claveria, Hibert Huaylla, Ana María Mejía-Jaramillo, Omar Triana-Chávez, Edison Osorio, Lorena Celina Luna, Gabriela E. Feresin

**Affiliations:** 1 Instituto de Biotecnología, Facultad de Ingeniería, Universidad Nacional de San Juan, Av. General San Martin 1109 (Oeste), San Juan 5400, Argentina; olimpiallcr@gmail.com (O.L.-C.); jortiz@unsj.edu.ar (J.E.O.); mauridpg@gmail.com (M.P.); lorenaluna@unsj-cuim.edu.ar (L.C.L.); 2 Consejo Nacional de Ciencia y Tecnología (CONICET CCT San Juan), Av. General San Martin 1109 (Oeste), San Juan 5400, Argentina; 3 Escuela Profesional de Ingeniería Agroindustrial, Universidad Nacional de Moquegua, Prolongación Calle Ancash s/n, Moquegua 18001, Peru; 4 Departament de Biologia, Sanitat i Medi Ambient, Facultat de Farmàcia i Ciències de l’Alimentació, Universitat de Barcelona, Av. Joan XXIII 27-31, 08028 Barcelona, Spain; ruscheltallini@ub.edu (L.R.T.); lauratorrascl@ub.edu (L.T.-C.); 5 Herbario del Sur de Bolivia, Instituto de Botánica y Ecología, Universidad Mayor Real y Pontificia de San Francisco Xavier de Chuquisaca, Calle Junín Esq. Estudiantes 692, Sucre, Bolivia; hiberhuaylla@gmail.com; 6 Grupo Biología y Control de Enfermedades Infecciosas—BCEI, Universidad de Antioquia UdeA, Calle 70 No. 52-21, Medellín 050010, Colombia; maria.mejia3@udea.edu.co (A.M.M.-J.); omar.triana@udea.edu.co (O.T.-C.); 7 Grupo de Investigación en Sustancias Bioactivas GISB, Facultad de Ciencias Farmacéuticas y Alimentarias, Universidad de Antioquia UdeA, Calle 70 No. 52-21, Medellín 050010, Colombia; edison.osorio@udea.edu.co

**Keywords:** Amaryllidaceae alkaloids, *Eustephia*, Cholinesterases, *Trypanosoma cruzi*

## Abstract

The Amaryllidaceae family represents a prolific source of pharmacologically active compounds, boasting over 700 diverse alkaloids identified to date. However, the genus *Eustephia* (Amaryllidoideae subfamily) remains largely unexplored. This study focused on the alkaloid profiles and pharmacological potential of bulb and leaves extracts from three Peruvian *Eustephia* species (*E. coccinea*, *E. darwinii*, and *E. hugoei*). The phenolic and flavonoid levels as well as the antioxidant activity of the methanolic extracts, were determined. Twenty-six alkaloids were identified in the alkaloid-enriched extracts (AEEs). Homolycorine-type alkaloids predominated in *E. darwinii* and *E. hugoei*, whereas *E. coccinea* displayed greater chemical diversity showing assoanine as the main detected alkaloid. In addition, candimine was widely distributed across species. AEEs showed stronger enzyme inhibition of acetylcholinesterase (AChE) compared to butyrylcholinesterase (BuChE). Notably, the AEE from *E. coccinea* leaves showed the highest AChE inhibition (IC_50_ = 1.82 μg/mL), while the AEE from bulbs exhibited the strongest BuChE inhibitory activity (IC_50_ = 61.22 μg/mL). Regarding anti-*T. cruzi* effect, the *E. darwinii* bulbs AEE was most potent and selective against amastigote forms (IC_50_ = 2.1 μg/mL; SI = 8.83). These findings underscore the potential of Peruvian *Eustephia* species as promising sources of pharmacologically relevant alkaloids, with possible applications in neurodegenerative disorders and Chagas disease.

## 1. Introduction

Natural products have always been a key source for discovering therapeutic agents, due to their remarkable structural diversity and broad spectrum of biological activities [1]. The Amaryllidaceae family, particularly the subfamily Amaryllidoideae, is notable for producing a unique and diverse range of alkaloids known as Amaryllidaceae alkaloids [2], boasting over 700 diverse alkaloids have been identified to date [3], many of which display pharmacological properties including anticancer, antiviral, antibacterial, anti-inflammatory effects, and the inhibition of acetylcholinesterase (AChE) and butyrylcholinesterase (BuChE). Among them, galanthamine (Gal) has been clinically approved for the palliative treatment of Alzheimer’s disease [4]. Chagas disease (CD), caused by *Trypanosoma cruzi*, affects over 7 million people globally, mainly in Latin America. While traditionally spread by insect vectors in rural areas, urban migration has shifted the risk to cities, even where transmission is no longer active. CD now spreads through non-vector routes like congenital transmission, blood transfusions, and organ transplants. Despite its global reach, treatment remains limited and available drugs (benznidazol and nifurtimox) are toxic, less effective in chronic cases, and often inaccessible [5,6,7]. Multiple alkaloids derived from the Amaryllidaceae family have demonstrated potent in vitro trypanocidal activity, notably candimine and hippeastrine which exhibit selective efficacy against the intracellular amastigotes form. These findings underscore the therapeutic potential of Amaryllidaceae alkaloids as promising leads in the development of novel antiparasitic agents [8,9,10]. In contrast, non-alkaloidal secondary metabolites, such as phenolics and flavonoids known for their antioxidant activity, remain comparatively underexplored within the Amaryllidoideae subfamily. To date only 224 of them has been identified, representing approximately 7% of the subfamily chemical diversity [11].

The genus *Eustephia* Cav. (Amaryllidaceae, subfamily Amaryllidoideae), native to the Andean regions of Peru and Bolivia, represents a small group and belongs to the tribe *Eustephieae* together with *Pyrolirion* Herb., *Hieronymiella* Pax., and *Chlidanthus* Herb. [12]. Phylogenetically, *Eustephia* is distinguished by the absence of an ITS2 indel typical of other Andean clade members, reflecting its unique evolutionary history shaped by Andean orogeny [12]. According to the World Checklist of Selected Plant Families (WCSP), the genus comprises six species: *E. armifera* J. F. Macbr., *E. coccinea* Cav., *E. darwinii* Vargas, *E. hugoei* Vargas, *E. kawidei* Vargas, and *E. longibracteata* Vargas [13].

In Peru, species of the the genus *Eustephia* are predominantly distributed in southern regions, where they are locally known by names such as “*campanilla*”, “*uluipiña*” [14], and “*para t’ika*” (Quechua, “rain flower”), reflecting the traditional belief that their blooming signals the onset of the rainy season. These plants are typically found in *rupicolas* and *saxicolous* habitats along the eastern of the Andean slopes, especially in upper inter-Andean valleys with seasonally dry to subhumid shrub–herb vegetation. Despite their cultural and ecological relevance, scientific studies into *Eustephia* remain limited. Only *E. coccinea* ethanolic extract (traditionally employed in the treatment of inflammatory conditions) was reported as active against *Staphylococcus aureus* [15].

This study represents the first comprehensive research into the phytochemical composition and pharmacological potential of *Eustephia* species, specifically *E. coccinea*, *E. darwinii*, and *E. hugoei*, native from the southern Peruvian Andes. By characterizing their alkaloid profiles, quantifying total phenolics and flavonoids, and evaluating antioxidant activity, cholinesterase inhibition (AChE and BuChE), and in vitro anti-*T. cruzi* effects, this work not only fills a significant gap in the phytochemical knowledge of a culturally and ecologically important genus, but also identifies promising bioactive compounds with potential applications in the treatment of neurodegenerative and parasitic diseases. These findings underscore the value of underexplored Andean flora as a reservoir of novel therapeutic agents and provide a scientific foundation for future drug discovery and conservation efforts.

## 2. Results and Discussion

### 2.1. Alkaloid Profile by GC-MS and UPLC-MS/MS

**Figure 1:**
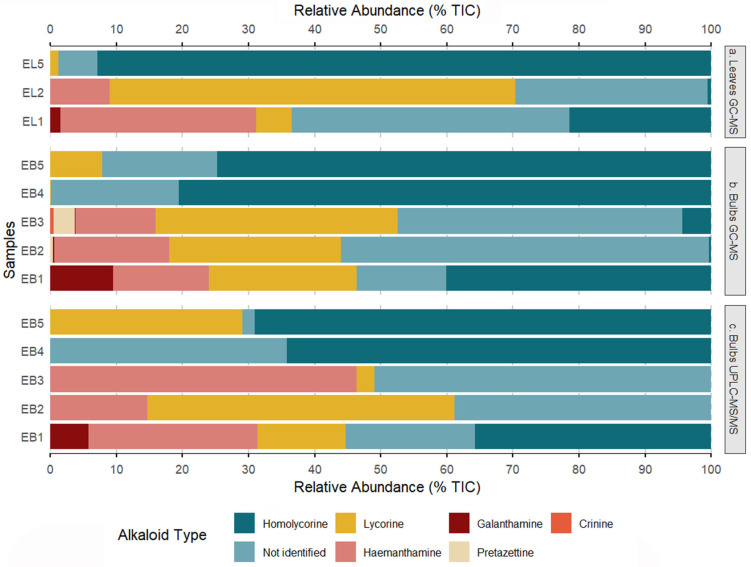
Alkaloid Profile of *Eustephia* by GC-MS and UPLC-MS/MS. Bars show the within-sample relative alkaloid composition, derived from the %TIC signal and normalized to 100%. The analysis is presented across three profiles: (**a**) Leaves GC-MS, (**b**) Bulbs GC-MS and (**c**) Bulbs UPLC-MS/MS. Sample codes: EB = bulbs, EL = leaves. EB1/EL1 (*E. coccinea*, Taray); EB2/EL2 (*E. coccinea*, Pisac); EB3 (*E. coccinea*, Tinta); EB4 (*E. darwinii*); EB5/EL5 (*E. hugoei*).

**Figure 2:**
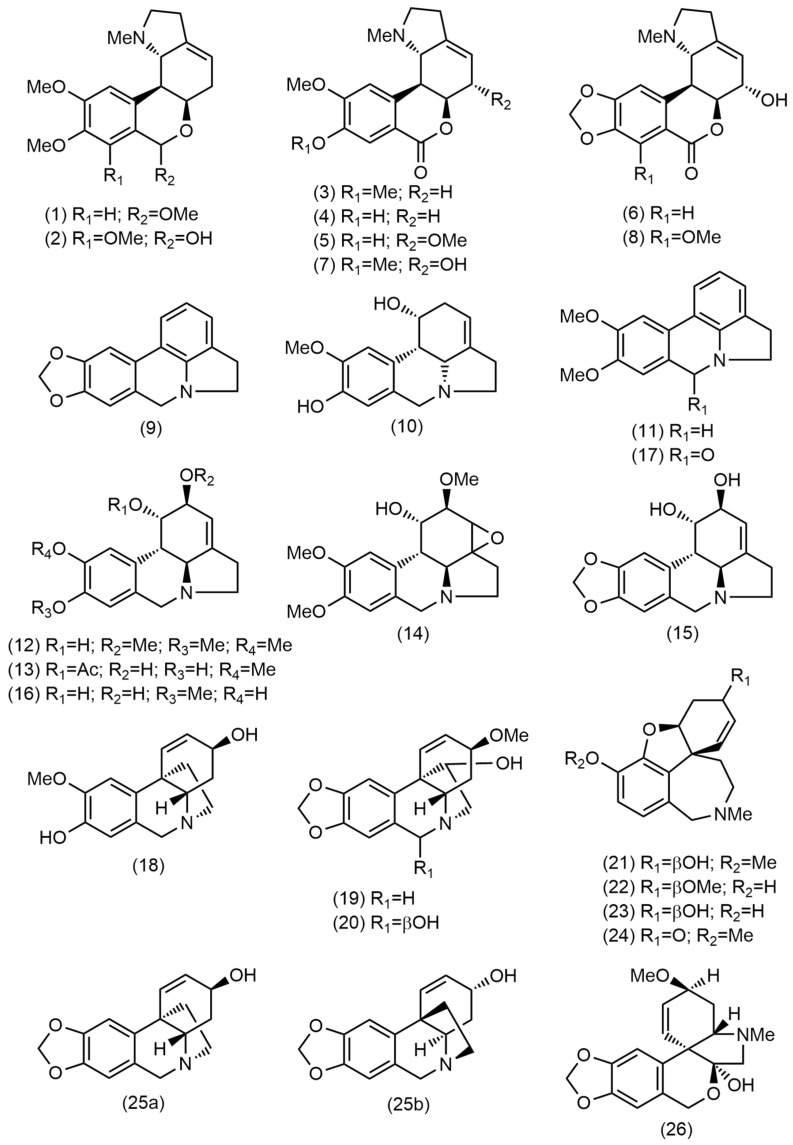
Chemical structures of the identified alkaloids in Peruvian *Eustephia* species.

Alkaloid profiling was conducted on alkaloid-enriched extracts (AEEs) obtained from both the bulbs and leaves of three *Eustephia* species: *E. coccinea* (from three locations), *E. hugoei*, and *E. darwinii* (sample codes in Table 1). GC-MS analysis revealed the presence of approximately sixty-three alkaloids in the analyzed samples. Twenty-six of them were successfully identified (Table 1, Figure 1 and Figure 2); and those components contributing less than 5% of the total ion current (TIC), were excluded. UPLC-MS/MS analysis of bulb samples provided complementary structural information. GC-MS spectra of the identified alkaloids as well as UPLC-ESI-MS/MS data of *Eustephia* species alkaloid extracts, were included in Supplementary Material (Tables S1–S6).

Homolycorine-type alkaloids predominated across the overall profile, followed by not identified compounds and lycorine-type alkaloids (Figure 1a,b). Although the alkaloids types detected were consistent between leaves and bulbs, their relative distribution (% TIC) showed differential accumulation patterns. In leaf sample EL2, lycorine represented the highest proportion (>60% TIC), while in EL5 homolycorine was the predominant alkaloid. While, bulbs samples showed greater heterogeneity in their compositional patterns. For instance, EB1 and EB3 displayed mixed profiles with relatively balanced contributions from multiple alkaloid types, while EB4 and EB5 exhibited marked specialization toward the homolycorine type alkaloids, exceeding 70% of their relative composition GC-MS profile.

A comparative analysis of the three *E. coccinea* populations revealed distinct chemotypes variations influenced by both geographic origin (locality) and plant organ. EB1 (bulb) displayed diverse chemical profile; with haemanthamine, candimine, hippeastrine, and Gal contributing between 7.8% and 14.5% of TIC.

Conversely, EL1 alkaloid profile showed high content of the non-identified compound NI-2 (*m*/*z* 301; 40.1% TIC), haemanthamine (25.7% TIC), and candimine (19% TIC). Samples EB2 and EL2 exhibited a relatively simple alkaloid profile enriched in lycorine-type constituents, assoanine accounting for 25.9% and 54.4% TIC; and NI-2 (*m*/*z* 301) for 16.4% and 9.2% TIC in bulb and leaves extracts, respectively. In contrast, EB3 displayed a markedly different chemical composition, characterized by sternbergine dominance (33.8% TIC) and a substantial presence of NI-2 (*m*/*z* 301; 39.3% TIC). *E. coccinea*, *E. darwinii* (EB4), and *E. hugoei* (EB5/EL5) exhibited clearly distinct alkaloid profiles. EB4 was primarily composed of 2-hydroxyhomolycorine (24.8% TIC) and 8-*O*-Demethylhomolycorine (22.7% TIC), and, in a lesser extent, candimine and hippeastrine. Regarding *E. hugoei*, both bulb and leaf samples (EB5 and EL5), were mainly characterized by 8-*O*-Demethylhomolycorine (46.5% and 61.8% TIC, respectively), followed by candimine as the second most abundant alkaloid (16.0% and 15.9% TIC).

The differences observed between bulbs and leaves of *E. coccinea* align with patterns reported in other Amaryllidaceae species. For example, in *Zephyranthes candida* (Lindl.) Herb., chemically distinct profiles have been reported between bulbs and leaves, with selective accumulation of alkaloids such as Gal and lycorine, which Xie et al. (2025) [16] attribute to organ-dependent regulation of biosynthetic genes depending on the organ analyzed. Similarly, studies on *Zephyranthes fosteri* also reveal distinct alkaloid profiles between bulbs and leaves, with lycorine as a characteristic of the former [17]. In addition to the differences between organs, the results reveal geographical variability within the species. However, despite this variation, the same compound NI-2 (*m*/*z* 301), was recurrently detected, appearing as a common element among the analyzed individuals, although its proportion was not constant. Such intraspecific geographic differences in alkaloid profiles are in accordance with previous observations in Amaryllidaceae; in *Eustephieae* tribe, distinct profiles reported for *Hieronymiella marginata* (Pax) Hunz. and *H. clidanthoides* Pax, between provinces in Argentina [18].

These differences reflect not only spatial and organs variation but also the phenological stage at the time sampling. Unlike the other populations, which were collected during vegetative growth, EB3 was collected during flowering phase. Previous studies have shown that the developmental stage plays a critical role in alkaloid biosynthesis within Amaryllidaceae, with both gene expression and metabolite accumulation exhibiting dynamic shifts across organs and growth phases [19]. Furthermore, the differential chemical profile would be related with edaphic conditions, such as soil pH, and nutrient availability, and rhizosphere microbiomes which could significantly modulate alkaloid profiles in plants [20].

UPLC-MS/MS analysis of bulb extracts was conducted to complement the GC-MS profiling, leveraging its enhanced sensitivity for detecting non-volatile and thermally labile alkaloids. The UPLC-MS/MS analysis showed [M + H]^+^ ions corresponding to several alkaloids previously identified by GC-MS, including candimine, haemanthamine, galanthamine, and hippeastrine. Additionally, tentative signals consistent with assoanine (*m*/*z* 268) and sternbergine (*m*/*z* 290) were identified. However, significant variations in the alkaloids relative abundance were evident between the two analytical techniques. Furthermore, UPLC-MS/MS also detected [M + H]^+^ ions at *m*/*z* 266, 304, 320, 360, and 362, not detected in the GC-MS analysis, highlighting its complementary detection capabilities. Therefore, the integration of both analytical techniques (GC-MS and UPLC-MS/MS methodologies) offered a complete overview of the alkaloid composition in *Eustephia* species.

The observed chemotypic diversity in *Eustephia* species, reflects a complex interplay of genetic, environmental, geographical, and developmental factors. Especially, the recurrent presence of not identified compounds contributing significantly to the relative abundance across multiple *Eustephia* alkaloid extracts reinforces the value of these plants as promising sources of potentially novel alkaloids.

### 2.2. Cholinesterases Inhibitory Bioassay

The inhibitory activities of AChE and BuChE were evaluated for AEE from *E. coccinea*, *E. darwinii*, and *E. hugoei*. The results, expressed as IC_50_ values, are summarized in Table 2. All tested samples exhibited AChE inhibitory activity, with greater potency compared to BuChE. EB2 (*E. coccinea,* Pisac) showed the stronger AChE inhibition (IC_50_ of 2.89 μg/mL), while EB1 (*E. coccinea*, Taray), EB4 (*E. darwinii*), and EB5 (*E. hugoei*) also displayed notable AChE activity (IC_50_ values below 10 μg/mL). Regarding leaves extracts, EL2 (*E. coccinea*, Pisac) was the most potent among all samples (IC_50_ equal to 1.82 μg/mL). In contrast, EL1 (*E. coccinea*, Taray) and EL5 (*E. hugoei*) did not show relevant effect (IC_50_ values of 170.53 and 195.72 μg/mL, respectively). In regard to BuChE, only three out of the eight extracts showed inhibition. EB1 exhibited the strongest inhibitory activity (IC_50_ = 61.22 µg/mL), while EB3 and EL2 showed relatively weak inhibition.

The results presented in Table 2 are in accordance with the alkaloid profiles of the *Eustephia* samples. EB2 and EL2 exhibited inhibitory activity against AChE and comparatively weaker inhibition of BuChE, these samples were characterized by a high relative abundance of assoanine (See Table 1), a reported AChE inhibitor. The inhibitory potency of assoanine is attributed to its enhanced binding affinity, which is facilitated by the presence of an aromatic ring at position C, contributing to its interaction with the enzyme’s active site [21]. EB1 containing galanthamine-type alkaloids (including Gal), exhibited potent AChE and BuChE inhibition. In contrast, extracts from *E. darwinii* (EB4) and *E. hugoei* (EB5 and EL5), characterized by high homolycorine-type alkaloids content (e.g., candimine and 8-*O*-Demethylhomolycorine), exhibited weak cholinesterase inhibition.

### 2.3. Anti-T. cruzi Activity

The anti-*T. cruzi* activities of AEEs from *Eustephia* species were evaluated against both epimastigotes and intracellular amastigotes form, while cytotoxicity was assessed in Vero cells. The corresponding IC_50_ values and selectivity indices (SI) are summarized in Table 3. All tested AEEs exhibited trypanocidal activity, with varying potency according to the developmental stage of the parasite. These findings suggest differential efficacy across parasite forms and highlight the potential of *Eustephia* species and its alkaloids as candidates for further antiparasitic investigation.

Among bulbs extracts, *E. darwinii* (EB4) showed the highest efficacy and selectivity, inhibiting both epimastigotes (IC_50_ = 3.73 µg/mL) and amastigotes (IC_50_ = 2.1 µg/mL), exhibiting low cytotoxicity in Vero cells (IC_50_ = 18.55 µg/mL), yielding SI values of 4.97 (epimastigotes) and 8.83 (amastigotes). EB1 (*E. coccinea*, Taray) and EB3 (*E. coccinea*, Tinta), showed similar trypanocidal activity against epimastigotes (IC_50_ = 4.21 and 3.45 µg/mL, respectively), and amastigotes (IC_50_ = 2.7 and 1.69 µg/mL, respectively). However, their higher cytotoxicity in Vero cells (IC_50_ = 13.59 and 3.69 µg/mL, respectively) resulted in lower selectivity (SI ≤ 5.18). EB2 (*E. coccinea*, Pisac) and EB5 (*E. hugoei*) exhibited moderate activity, with IC_50_ values ranging from 5.58–5.73 µg/mL against amastigotes, and intermediate SI values (2.52–3.01).

Leaves extracts exhibited weak activity, requiring higher concentrations to inhibit parasite growth. EL1 (*E. coccinea*, Taray) and EL5 (*E. hugoei*) showed poor activity (IC_50_ > 28 µg/mL in amastigotes) and low selectivity (SI ≤ 2.92). In contrast, EL2 (*E. coccinea*, Pisac) exhibited comparatively better activity (IC_50_ = 9.01 µg/mL against amastigotes) and an SI of 6.15, indicating moderate selectivity.

Overall, these results indicate that bulb extracts were generally more effective than leaves extracts, being EB4 (*E. darwinii*) the most selective and potent sample against *T. cruzi* intracellular amastigotes, the clinically relevant stage of the parasite. The remarkable selectivity of EB4 even surpassed that of the reference drug benznidazole (SI = 3.01 and 2.22). This enhanced activity may be due to the higher concentration of the alkaloids hippeastrine and candimine, both of which have previously been reported as effective against *T. cruzi* [10,22].

### 2.4. Total Phenolic Content (TPC), Flavonoid Content (FC), and Antioxidant Activity

Phenolic compounds, particularly flavonoids, have garnered significant attention for their neuroprotective properties. These plant-derived molecules exhibit potent antioxidant activity, which helps mitigate oxidative stress, a key contributor to neurodegenerative diseases such as Alzheimer’s and Parkinson’s. Flavonoids can scavenge reactive oxygen species (ROS), modulate signaling pathways involved in neuronal survival, and inhibit neuroinflammation [23]. Additionally, some flavonoids have demonstrated the ability to inhibit enzymes like acetylcholinesterase, further supporting their role in preserving cognitive function. Their multifunctional bioactivity position them as promising candidates for the development of therapeutic agents targeting neurodegeneration [24].

Table 4 presents the total phenolic content (TPC), flavonoid content (FC), and antioxidant activity (DPPH, ABTS, and FRAP) of bulb methanolic extracts (BMEs) from five *Eustephia* bulbs samples. TPC varied significantly among the samples, with *E. coccinea* (EB3) and *E. hugoei* (EB5) showing the highest values (22.41 and 23.20 mg GAE/g, respectively). Compared to other studies, the TPC values are higher than those reported for bulbs of *Hieronymiella peruviana* (12.89 mg GAE/g) [25]. FC showed limited variation among samples with EB4 (*E. darwinii*) comprising the highest value (2.19 mg QE/g), significantly higher than EB1 (*E. coccinea*, Taray) and EB5 (*E. hugoei*), while EB2 (*E. coccinea*, Pisac) and EB3 were statistically similar to EB4. These values are comparable to those of other species, such as *Narcissus pseudonarcissus* (1.19 mg QE/g) [26]. This interspecific variation may be attributed to environmental stress, as plants growing at high altitudes face harsh conditions such as increased UV radiation and lower temperatures [27].

Antioxidant activity was not mirrored by TPC. EB4, despite having the lowest TPC (11.14 mg GAE/g), showed ABTS activity (19.54% inhibition at 1 mg BME/mL) similar to that of EB3 and EB5, which had higher TPC. Conversely, EB5, with high TPC, displayed relatively low DPPH activity (19.00% at 0.5 mg BME/mL). Quercetin was used as a reference compound and exhibited more than 90% radical scavenging activity against DPPH and ABTS radicals at 50 µg/mL and 3 µg/mL, respectively. FRAP remained statistically indistinguishable across all samples (3.35–3.45 mg TE/g), with no significant differences, despite variation in TPC, FC, and the antioxidant activities measured by the DPPH and ABTS assays. Similar patterns have been observed in other Amaryllidaceae species, such as *Sternbergia* spp., which exhibited generally weak FRAP activity despite high phenolic content [28], and *Crinum* spp., which showed broad variation in TPC and FC levels without consistently stronger DPPH or FRAP responses [29].

This apparent discrepancy between phenolic content and antioxidant efficacy can be attributed to the complex nature of antioxidant mechanisms and the different processes measured in the different assays. DPPH and ABTS assess radical-scavenging capacity, while FRAP specifically quantifies the reducing power through single-electron transfer. The uniformity of the FRAP results, despite the variations in the TPC could probably be due to the contribution of multiple non-phenolic reductants such as alkaloids, terpenoids, and vitamins. Given their presence in these extracts, it is plausible that these non-phenolic compounds act synergistically or additively with phenolics, resulting in a homogenous net reducing capacity that masks the underlying variability in phenolic levels.

## 3. Materials and Methods

### 3.1. Chemicals

All chemicals employed were of analytical grade. Methanol and Sulfuric acid (H_2_SO_4_) were purchased from Merck Química Argentina (Buenos Aires, Argentina). Hydrochloric acid was purchased from Biopack (Buenos Aires, Argentina). Commercial Folin-Ciocalteu (FC) reagent, 2,2-diphenyl-1-picrylhydrazyl (DPPH), ferric chloride hexahydrate, 2,4,6-tris(2-pyridyl)-s-triazine, trolox (T), quercetin (Q), gallic acid (GA), AChE from *Electrophorus electricus* (electric eel), BuChE from equine serum, potassium phosphate (K_2_HPO_4_), sodium dihydrogen phosphate (NaH_2_PO_4_), sodium dihydrogen phosphate (NaH_2_PO_4_), sodium chloride (NaCl), 5,5′-dithio-bis-(2-nitrobenzoic acid) (DTNB), acetylthiocholine iodide (ATC), butyrylthiocholine iodide (BTC) and Gal were obtained from Sigma-Aldrich, St. Louis, MO, USA. The software Prism 10.4.1 (GraphPad Software Inc., San Diego, CA, USA) was used.

### 3.2. Plant Material

**Figure 3:**
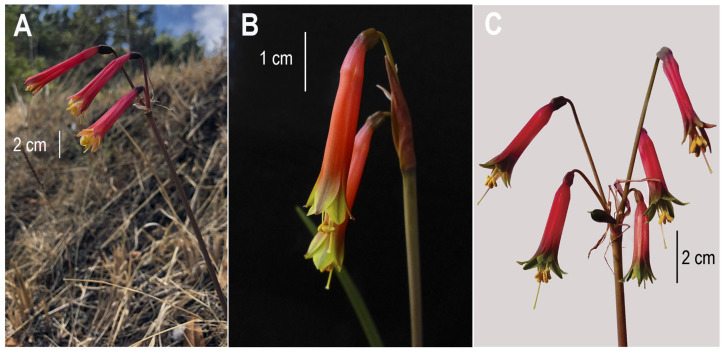
Representative photographs of *Eustephia* species. (**A**) *E. coccinea*; (**B**) *E. darwinii*; (**C**) *E. hugoei*. Pictures source: (**A**) Olimpia Llalla-Cordova; (**B**,**C**) Hibert Huaylla.

Samples were collected in the Cusco and Apurímac regions. In Cusco, *E. coccinea* was sampled at Taray (13°26′49.8″ S 71°52′17.8″ W), Pisac (13°25′13.3″ S 71°50′46.9″ W) and Tinta (14°07′23.1″ S 71°24′32.4″ W); *E. darwinii* at Circa (13°52′13.9″ S 72°56′52.3″ W); and *E. hugoei* at Lambrama (13°49′42.6″ S 72°47′35.2″ W). Representative flowers are shown in Figure 3. The specimens were identified by botanic Hibert Huaylla and deposited in the Herbarium MOQ (Universidad Nacional de Moquegua, Moquegua, Peru) and LIL (Universidad Nacional de Tucuman, Tucuman, Argentina). The voucher details are as follows: *E. coccinea* specimens from Taray (MOQ 2026; LIL 618678), Pisac (MOQ 2027; LIL 618679), and Tinta (MOQ 2030; LIL 618682); *E. darwinii* (MOQ 2029; LIL 618681); and *E. hugoei* (MOQ 2028; LIL 618680).

Table 5, provides plant part and phenological stage. The temperatures recorded in the habitats of these species during the wet season (November–April) range from 5 °C to 18 °C, coinciding with the highest annual precipitation (600–775 mm). In contrast, the dry season (May–October) is characterized by a wide thermal range (−5 °C to 24 °C) and scarce rainfall. The mean relative humidity ranged between 48% and 56% [30].

The studied material, is characterized by ovoid to oblong bulbs that produce small bulblets for propagation. The linear leaves emerge after the flowering. All three species possess a basally fused perianth tube. *E. coccinea* (flowers 3.3–3.8 cm long) has uniformly scarlet outer tepals extending to the apex, with stamens approximately equal in length to the tepals. *E. darwinii* (4–5 cm long) is characterized by outer tepals with undulate, greenish-yellow apices and stamens that exceeding their length. *E. hugoei* (approximately 5 cm long) is distinguished by outer tepals ending in flat, dark green apex.

### 3.3. Extraction

The bulbs and leaves were dried under an air current at 40 °C until they reached a constant weight, then macerated in MeOH for 72 h, the volume of MeOH varied according to the amount of sample. The solvent was evaporated using a rotary evaporator, resulting in the methanolic extract. A portion of the bulb methanolic extract (BME) was reserved for analysis of TPC, FC, and antioxidant activity. Yields are expressed as % (*w*/*w*) of dry plant material (Table 4).

In order to obtain the AEEs, the methanolic extracts were then acidified to pH 3.5 with H_2_SO_4_ (2%, *v*/*v*). Subsequently, the neutral material was removed with Et_2_O (3 × 100 mL). The remaining aqueous phase was alkalinized to pH 9–10 with 20% (*w*/*v*) NaOH and subjected to extractions with AcOEt (3 × 100 mL) to recover the alkaloids. The basic AcOEt solution was separated and anhydrous sodium sulphate was added to remove any remaining water. Then, it was concentrated in a rotary evaporator until dryness, obtaining the alkaloid-enriched extract (AEE). Yields are expressed as % (*w*/*w*) of dry plant material (Table 2).

### 3.4. Gas Chromatography Coupled to Mass Spectrometry (GC-MS) and Ultra Performance Liquid Chromatography Tandem Mass Spectrometry (UPLC-MS/MS) Analysis

The AEE of *Eustephia* species were analyzed by GC-MS, using an equipment model 6890/MSD 5975 (Hewlett Packard, Palo Alto, CA, USA), operating in electron impact ionization mode (70 eV). Compound separation was carried out with a DB-5 MS column (30 m × 0.25 mm × 0.25 µm). The temperature program was as follows: from 100 °C to 180 °C at a rate of 15 °C/min, with a 1-min hold at 180 °C; followed by an increase from 180 °C to 300 °C at 5 °C/min, maintaining this temperature for 1 min. The injector temperature was set at 280 °C, with helium flow as carrier gas at 0.8 mL/min and a split ratio of 1:20.

The data were processed with AMDIS 2.64 software (NIST) and compared with a private Amaryllidaceae alkaloid library and literature reports. Compounds were identified by comparing mass spectra and Kovats retention index (RI) values with previously characterized Amaryllidaceae alkaloids at the Natural Products Laboratory, University of Barcelona. The NIST 05 database and additional literature references were also consulted. Relative abundance was expressed as a percentage of the total ionic current (TIC).

The LC-MS analysis was performed in an ACQUITY H–Class UPLC instrument equipped with a XEVO TQ-S micro triple quadrupole mass spectrometer (Waters Corp., Milford, MA, USA) with electrospray ionization (ESI). An UPLC ACQUITY BEH C18 (1.7 μm, 2.1 mm × 100 mm) column was used for separation at 35 °C. The mobile phase con-sisted of A (0.1% formic acid), B (acetonitrile, 0.1% formic acid), and C (methanol) with a flow rate of 0.2 mL/min. The gradient conditions were as follows: initially, 95%A–5%B and hold for 2 min; 5 min, 85%A–15%B; 10 min, 80%A–10%B–10%C and hold for 4 min; 17 min, 60%A–20%B–20%C; 18 min, 95%A–5%B and hold for 2 min; completing 20 min. The AEEs samples were prepared at 100 ppm. The samples were dissolved in a mixture of methanol:water (50:50) and filtered through a membrane filter (0.22 µm). The injection volume was 10 µL. The capillary, cone, and collision energies were 2 kV, 43 V, and 30 eV, respectively. The data were acquired in ESI positive mode, MS2 scan function (50–1000 Da), and processed using MassLynx Software V4.2 (Waters, Milford, MA, USA). The combination of these techniques enabled a comprehensive qualitative and semi-quantitative profiling of the metabolites in the samples, facilitating the precise identification of the alkaloids of interest.

### 3.5. AChE and BuChE Inhibition Assays

The inhibitory activity of AChE and BuChE enzymes was evaluated following the method of Ellman et al. (1961) [31] with modifications. In each well of a 96-well microplate, 50 µL of AChE or BuChE enzyme solution (0.25 U/mL, in phosphate buffer: 8 mM K_2_HPO_4_, 2.3 mM NaH_2_PO_4_, 0.15 M NaCl, pH 7.5) and 50 µL of the extract dissolved in the same buffer were added. The plates were incubated at room temperature for 30 min. Subsequently, 100 µL of the substrate solution, containing DTNB and ATC or BTC (0.6 mM), prepared in a saline solution with Na_2_HPO_4_ (pH 7.5), was added. The absorbance at 405 nm was recorded using a Thermo Scientific Multiskan FC spectrophotometer (Waltham, MA, USA), 5 min after the reaction began. The concentrations assayed ranged from 1 to 200 µg/mL. Gal was used as a positive control. IC_50_ values were expressed as the mean ± standard deviation (SD) of three individual determinations, each performed in triplicate.

### 3.6. In Vitro Trypanocidal Activity Assay

*T. cruzi* Tulahuen-β-galactosidase parasites (DTU VI) were cultured as epimastigotes at 28 °C in liver infusion tryptose (LIT) medium supplemented with 10% (*v*/*v*) heat-inactivated fetal bovine serum (FBS). For assays involving intracellular stages, Vero cells were used as host cells and maintained in DMEM supplemented with 2% FBS and 1% P-S-G.

#### 3.6.1. Anti-Epimastigote Assay

Test plates were prepared using initial extract concentrations of 400 µg/mL, which were serially diluted 1:2 to generate a concentration-response curve. Benznidazole (Bzn) was included as the positive control. Plates were incubated at 28 °C for 4 days. Subsequently, 50 µL of PBS containing 10% alamarBlue reagent was added to each well, and plates were incubated overnight at 28 °C prior to measuring fluorescence intensity (excitation: 530 nm, emission: 590 nm). All assays were performed at least in triplicate.

#### 3.6.2. Anti-Amastigote Assay

This assay was performed following the protocol described by Ortiz et al. (2023) [22]. Briefly, 5 × 10^6^ Vero cells were seeded in a T-175 flask and incubated for 24 h. Cells were then infected with 1 × 10^7^ trypomastigotes (MOI~1) for 18 h. After infection, the monolayers were washed with PBS, detached, and diluted to 5 × 10^5^ cells/mL. Aliquots of 100 µL were added per well to plates containing the extracts. Bzn was used as a control. After 4 days at 37 °C, plates were frozen at −80 °C to stop the assay. Then, 50 µL per well of PBS containing 0.25% NP-40 and 500 µM chlorophenol red-β-d-galactoside (CPRG) substrate was added, and plates were incubated for an additional 4 h at 37 °C before measuring absorbance at 590 nm. All assays were performed at least in triplicate.

#### 3.6.3. Vero Cell Cytotoxicity Assay

Vero cell toxicity was evaluated as described by Ortiz et al. (2023) [22]. Cells were diluted to 5 × 10^5^ cells/mL, and 100 µL per well were added to plates containing the extracts. Each plate included appropriate negative and positive controls. Plates were incubated at 37 °C for 4 days, after which 50 µL per well of PBS containing 10% alamarBlue reagent was added. Plates were incubated for 6 h at 37 °C before measuring fluorescence (excitation: 530 nm, emission: 590 nm). All assays were performed at least in triplicate.

### 3.7. Total Phenolic Content (TPC) and Total Flavonoid Content (FC)

TPC was determined using the method described by Helrich et al. (1990) [32]. In a 96-well microplate, 10 µL of BME, 12.5 µL of diluted Folin-Ciocalteu reagent, and 37.5 µL of 20% (*w*/*v*) Na_2_CO_3_ were added. The reaction mixture was incubated at room temperature in the dark for 30 min, and the absorbance was subsequently measured at 750 nm using a Multiskan FC microplate reader (Thermo Scientific, USA). The calibration curve was constructed using gallic acid at concentrations of 0, 0.15, 0.3, 0.6, 1.2, and 2.35 mM. Results were expressed as milligrams of gallic acid equivalents per gram of methanolic extract (mg GAE/g BME).

The trichloride aluminum (AlCl_3_) colorimetric method was employed to assess the FC, based on the protocol by Ismail et al. (2010) [33]. In each well, 125 µL of BME and 125 µL of 2% (*w*/*v*) AlCl_3_ were added. The solution was left to stand for 10 min at room temperature. Absorbance was measured at 450 nm using a microplate reader (Thermo Scientific, USA). The calibration curve was constructed using quercetin standard solutions at concentrations of 0, 0.03, 0.07, 0.15, 0.22, and 0.30 mM. Results were expressed as milligrams of quercetin equivalents per gram of methanolic extract (mg QE/g ME). Values obtained in triplicate for TPC and FC are reported as mean ± standard deviation (SD).

### 3.8. Antioxidant Assays

#### 3.8.1. Radical-Scavenging Activity (DPPH)

Antioxidant capacity assessed by the DPPH radical-scavenging assay [34,35]. BME (1–500 µg/mL) incubated with DPPH (150 µL) in 96-well plates for 5 min in the dark at ambient temperature. Absorbance read at 517 nm (Multiskan FC, Thermo Scientific, Waltham, MA, USA). Quercetin (20–120 µg/mL) was used as reference standard. The percentage of discoloration (free radical scavenging capacity) was calculated using the following formula:

DPPH scavenging capacity (%) = [1 − ((A_s_ − A_c_)/A_DPPH_)] × 100 (1)

where A_c_ represents the control absorbance, A_s_ represents the absorbance of the tested extract, and A_DPPH_ the absorbance of DPPH radical.

#### 3.8.2. Ferric Reducing Antioxidant Power (FRAP)

FRAP was determined according to Benzie and Strain [36]. Briefly, the FRAP solution was freshly prepared by mixing acetate buffer 300 mM at pH 3.6, ferric chloride hexahydrate 20 mM dissolved in distilled water, and 2,4,6-tris(2-pyridyl)-s-triazine 10 mM dissolved in HCl 40 mM (10:1:1, *v*/*v*/*v*). Ten microliters of sample solution (1–500 µg mL^−1^) or Trolox (0–1 mM) were mixed with 190 μL of the FRAP solution in 96-well microplates in triplicate, solvent blanks included. The change in absorbance was measured at 595 nm (Multiskan FC, Thermo Scientific, Waltham, MA, USA). Results expressed as mg Trolox equivalents per g of BME (mg TE/g BME).

#### 3.8.3. Radical-Cation Decolorization Assay (ABTS)

The assay was conducted as described by Re et al. [37]. ABTS•^+^ prepared and diluted in PBS to A_734_ = 0.70. Serial dilutions of BME (1–500 µg/mL) dispensed in 96-well plates; 10 µL BME or Trolox combined with 200 µL ABTS•^+^. Incubation 4 min at 30 °C in the dark. Absorbance measured at 734 nm (Multiskan FC, Thermo Scientific, Waltham, MA, USA). The concentration of BME required to reach 50% scavenging activity was determined from inhibition–concentration curves. Results were expressed as % inhibition at 1 mg BME/mL.

### 3.9. Statistical Data Analysis

The AChE and BuChE inhibition assays were performed in triplicate, and IC_50_ values were expressed as the mean ± standard deviation (SD) from three independent experiments. Data were analyzed using Prism 10.4.1 (GraphPad Software Inc., San Diego, CA, USA). Total phenolic content (TPC), flavonoid content (FC), DPPH, ABTS and FRAP were analyzed by one-way ANOVA followed by Tukey’s HSD (Tukey-adjusted *p* < 0.05) in R 4.4.1; model assumptions (normality and homoscedasticity) were verified. Anti-*T. cruzi* IC_50_ values were analyzed using the Kruskal–Wallis test followed by Dunn’s post-hoc test with Benjamini–Hochberg adjustment (BH-adjusted *p* < 0.05).

## 4. Conclusions

This study provides the first comprehensive insight into the genus *Eustephia* as a promising and underexplored source of pharmacologically active alkaloids. The observed chemical diversity, driven by the plant parts and geographic location, highlights the genus’s chemotaxonomic complexity, particularly the abundance of homolycorine-type and non-identified alkaloids, being pharmacologically relevant assoanine, hippeastrine, candimine, and galanthamine. AEE of *E. darwinii* and *E. coccinea* exhibited potent AChE inhibition and selective anti-*T. cruzi* activity, positioning these species as promising candidates for future drug discovery. Additionally, *E. hugoei* stands out due to its distinctive alkaloid profile, which includes several unidentified compounds, suggesting the presence of novel bioactive constituents that merit further research, reinforcing the broader potential of the genus for discovering of new chemical compounds. Future studies should aim to isolate and elucidate the structures of these alkaloids and to further evaluate their biological activities, including in silico analyses of their mechanisms of action related to key enzymes (trypanothione reductase, trans-sialidase) as well as parasitemia reduction studies in mouse models.

## Figures and Tables

**Table 1:** GC-MS data for *Eustephia* species alkaloid extracts collected in Peru. Values expressed in %TIC (total ion current).

Alkaloids	[M+]	Ion Base	RI	EB1	EB2	EB3	EB4	EB5	EL1	EL2	EL5
**Homolycorine-type**											
*O*-Methyllycorenine (**1**)	331	109	2483.4				0.2				
Nerinine (**2**)	347	109	2515.6				3.9	1.3			
Homolycorine (**3**)	315	109	2765.5			0.2	0.4	4.1			7.4
8-*O*-Demethylhomolycorine (**4**)	301	109	2794.8	0.9			22.7	46.5			61.8
2-Methoxy-8-*O*-Demethylhomolycorine (**5**)	331	139	2825.1		0.3						
Hippeastrine (**6**)	315	125	2867.6	10.7		0.8	13.1				
2-Hydroxyhomolycorine (**7**)	331	125	2978.2	13.6		0.8	24.8	3.9		0.5	7.8
Candimine (**8**)	345	125	3078.4	13.6			15.4	16	19		15.9
**Lycorine-type**											
Anhydrolycorine (**9**)	251	250	2521.2	0.1			0.1				
Kirkine (**10**)	273	252	2573.4					1.1			
Assoanine (**11**)	267	266	2578.4	1.2	25.9				0.5	54.4	1.2
Galanthine (**12**)	317	242	2705.2	19.1		1.8			4.5		
Sternbergine (**13**)	331	228	2710.9	1.3		33.8					
Incartine (**14**)	333	332	2761.6	0.2							
Lycorine (**15**)	287	226	2767.6	0.5							
Pseudolycorine (**16**)	289	228	2823.0			0.9					
Oxoassoanine (**17**)	281	281	2934.4						0.4	1.3	
**Haemanthamine-type**											
8-*O*-Demethylmaritidine (**18**)	273	273	2532.0			0.1			3.9	7.6	
Haemanthamine (**19**)	301	272	2641.0	14.5	17.4	11.6			25.7	0.6	
Haemanthidine (**20**)	317	115	2733.1			0.3					
**Galanthamine-type**											
Galanthamine (**21**)	287	286	2398.6	7.8		0.1			0.6		
Chlidanthine (**22**)	287	287	2404.0	0.4	0.2						
Sanguinine (**23**)	273	273	2415.9	0.3					0.9		
Narwedine (**24**)	285	284	2475.3	1							
**Other-type**											
Vittatine/crinine (**25a**/**25b**)	271	271	2476.7			0.5					
Tazettine (**26**)	331	247	2649.3		0.4	3.2					
**Not identified**											
NI-1 (**27**)	329	268	2521.0		22.3			8.3		10.2	1.2
NI-2 (**28**)	301	301	2539.6	5.5	16.4	39.3			40.1	9.2	
NI-3 (**29**)	287	286	2556.8		11.9					5.9	
NI-4, lycorine-type (**30**)	327	266	2579.7					6.7			
Total alkaloid				90.7	94.8	93.4	80.6	87.9	95.6	89.7	95.3

Sample codes: EB = bulbs, EL = leaves. EB1/EL1 (*E. coccinea*, Taray); EB2/EL2 (*E. coccinea*, Pisac); EB3 (*E. coccinea*, Tinta); EB4 (*E. darwinii*); EB5/EL5 (*E. hugoei*). RI = retention index.

**Table 2:** Percentage yield (%), and inhibitory activity of alkaloid extracts of *E. coccinea*, *E. darwinii*, and *E. hugoei* against AChE and BuChE, IC_50_ values expressed as μg/mL.

Species	Sample	Yield (%)	AChE	BuChE
Bulbs				
*E. coccinea* (Taray)	EB1	0.44	4.56 ± 0.0 ^d,e,f^	61.22 ± 4.40 ^d^
*E. coccinea* (Pisac)	EB2	0.17	2.89 ± 0.65 ^e,f^	>200 ^a^
*E. coccinea* (Tinta)	EB3	0.19	57.22 ± 1.03 ^c^	125.14 ± 4.27 ^c^
*E. darwinii*	EB4	0.65	7.47 ± 0.60 ^d,e^	>200 ^a^
*E. hugoei*	EB5	0.19	9.04 ± 0.49 ^d^	>200 ^b^
Leaves				
*E. coccinea* (Taray)	EL1	0.81	170.53 ± 5.32 ^b^	>200 ^b^
*E. coccinea* (Pisac)	EL2	0.37	1.82 ± 0.06 ^f^	180.33 ± 2.47 ^b,c^
*E. hugoei*	EL5	0.10	195.72 ± 1.21 ^a^	>200 ^b^
Galanthamine	Gal		0.39 ± 0.006	5.35 ± 0.15

Different letters indicate significant differences between samples according to the Tukey test (*p* < 0.05).

**Table 3:** Anti-*Trypanosoma cruzi* activity of AEEs from *Eustephia*: IC_50_ (µg/mL) on epimastigotes (Epis) and intracellular amastigotes (Amas), Vero cytotoxicity, and selectivity index (SI).

Species	Sample	IC_50_ (µg/mL)			SI	
		Epis	Amas	Vero	Epis	Amas
Bulbs						
*E. coccinea* (Taray)	EB1	4.21 ± 0.003 ^b^	2.7 ± 0.006 ^b^	13.59 ± 0.01 ^b^	3.22	5.03
*E. coccinea* (Pisac)	EB2	17.52 ± 0.02 ^d^	5.58 ± 0.02 ^c^	14.08 ± 0.001 ^b^	0.80	2.52
*E. coccinea* (Tinta)	EB3	3.45 ± 0.002 ^a^	1.69 ± 0.001 ^a^	3.692 ± 0.01 ^a^	1.07	2.19
*E. darwinii*	EB4	3.73 ± 0.002 ^a^	2.1 ± 0.002 ^a^	18.55 ± 0.004 ^c^	4.97	8.83
*E. hugoei*	EB5	8.93 ± 0.01 ^c^	5.73 ± 0.02 ^c^	17.24 ± 0.004 ^c^	1.93	3.01
Leaves						
*E. coccinea* (Taray)	EL1	39.35 ± 0.07 ^e^	28.02± 0.001 ^e^	63.61 ± 0.013 ^d^	1.62	2.27
*E. coccinea* (Pisac)	EL2	40.35 ± 0.04 ^e^	9.01 ± 0.004 ^d^	55.47 ± 0.02 ^d^	1.37	6.15
*E. hugoei*	EL5	68.20 ± 0.04 ^f^	40.39 ± 0.001 ^f^	117.8 ± 0.0003 ^e^	1.72	2.92
Benznidazole	Bzn	6.46 ± 0.001	8.74 ± 0.003	19.42 ± 0.11	3.01	2.22

Different letters indicate significant differences between bulbs and leaves samples (BH-adjusted *p* < 0.05).

**Table 4:** Yield and Total phenolic content (TPC), flavonoid content (FC), and antioxidant activities (DPPH, ABTS, FRAP) in methanolic extracts of *Eustephia* bulbs.

Sample	Yield %	TPC ^1^ (mg GAE/g)	FC ^2^ (mg QE/g)	DPPH ^3^ %	ABTS ^4^ %	FRAP ^5^ (mg TE/g)
EB1	21.21	11.30 ± 1.53 ^c^	1.89 ± 0.16 ^b^	21.21 ± 1.81 ^ab^	13.38 ± 2.12 ^b^	3.36 ± 0.08 ^a^
EB2	9.88	18.45 ± 2.15 ^a^	2.12 ± 0.03 ^ab^	19.26 ± 2.68 ^ab^	18.38 ± 1.49 ^ab^	3.35 ± 0.02 ^a^
EB3	13.96	22.41 ± 1.67 ^ab^	2.12 ± 0.08 ^ab^	22.85 ± 2.32 ^a^	20.83 ± 1.34 ^a^	3.45 ± 0.15 ^a^
EB4	10.58	11.14 ± 0.48 ^c^	2.19 ± 0.05 ^a^	17.22 ± 0.69 ^b^	19.54 ± 3.23 ^ab^	3.35 ± 0.05 ^a^
EB5	8.04	23.20 ± 1.92 ^b^	1.93 ± 0.06 ^b^	19.00 ± 0.20 ^ab^	20.79 ± 2.96 ^a^	3.35 ± 0.05 ^a^

^1^ TPC expressed as mg gallic acid per g of methanolic extract (BME). ^2^ FC expressed as mg quercetin per g of bulb methanolic extract (BME). ^3^ Antiradical DPPH activities are expressed as a percentage at 0.5 mg BME/mL concentration. ^4^ ABTS antiradical activity is expressed as a percentage inhibition at a concentration of 1 mg BME/mL. ^5^ FRAP expressed as gr trolox equivalents/g of bulb methanolic extract (BME). Different letters (a, b, c) within the same assay column indicate significant differences (*p* < 0.05).

**Table 5:** Collection details of *Eustephia* samples from Peru.

Species	Location	Part	Sample Code	Phenology	Altitude (m.a.s.l.)
*E. coccinea*	Taray—Cusco	Bulb and Leaves	EB1, EL1	Vegetative (March)	3 332
*E. coccinea*	Pisac—Cusco	Bulb and Leaves	EB2, EL2	Vegetative (March)	3 062
*E. coccinea*	Tinta—Cusco	Bulb	EB3	Flowering (September)	3 523
*E. darwinii*	Circa—Apurimac	Bulb	EB4	Vegetative (March)	2 200
*E. hugoei*	Lambrama—Apurimac	Bulb and leaves	EB5, EL5	Vegetative (March)	2 713

m.a.s.l.: meters above sea level.

## Data Availability

The original contributions presented in this study are included in the article. Further inquiries can be directed to the corresponding author.

## Supplementary Materials

The following supporting information can be downloaded at: https://www.mdpi.com/article/10.3390/plants14223510/s1, Table S1: UPLC-ESI-MS/MS data for *Eustephia hugoei* (EB5) alkaloid extract. Table S2: UPLC-ESI-MS/MS data for *Eustephia darwinii* (EB4) alkaloid extract. Table S3: UPLC-ESI-MS/MS data for *Eustephia coccinea* (EB3) (Tinta - Cusco) alkaloid extract. Table S4: UPLC-ESI-MS/MS data for *Eustephia coccinea* (EB2) (Pisac - Cusco) alkaloid extract. Table S5: UPLC-ESI-MS/MS data for *Eustephia coccinea* (EB1) (Taray - Cusco) alkaloid extract. Table S6: GC-MS spectra of the identified alkaloids in *Eustephia* species from Peru.
