# Supplementary material for: Pharmacological Potential of Peruvian Eustephia Species (Amaryllidaceae): Alkaloid Diversity, Cholinesterase Inhibition, and Anti-Trypanosoma cruzi Activity
Source: Plants (Basel). 2025 Nov 18;14(22):3510. doi: 10.3390/plants14223510 (PMC12656678; doi:10.3390/plants14223510)
Supplement: Supplementary file 1 [file plants-14-03510-s001.zip › plants-3948410-supplementary.pdf]

Supplementary materials

Pharmacological Potential of Peruvian *Eustephia* Species (Amaryllidaceae): Alkaloid Diversity, Cholinesterase Inhibition, and Anti-*Trypanosoma cruzi* Activity

Olimpia Llalla-Cordova [1,2,3], Javier E. Ortiz [1], Mauricio Piñeiro [1,2], Luciana R. Tallini [4], Laura Torras-Claveria [4], Hibert Huaylla [5], Ana María Mejía-Jaramillo [6], Omar Triana-Chávez [6], Edison Osorio [7], Lorena Luna [1] and Gabriela E. Feresin [1,3,*]

[1] Instituto de Biotecnología, Facultad de Ingeniería, Universidad Nacional de San Juan, Av. General San Martin 1109 (oeste), San Juan, CP 5400, Argentina; olimpiallcr@gmail.com, jortiz@unsj.edu.ar, lorenaluna@unsj-cuim.edu.ar, gferesin@unsj.edu.ar, mauridpg@gmail.com

[2] Consejo Nacional de Ciencia y Tecnología (CONICET CCT San Juan), Av. General San Martin 1109 (oeste), San Juan, CP 5400, Argentina, gferesin@unsj.edu.ar, olimpiallcr@gmail.com, mauridpg@gmail.com

[3] Escuela Profesional de Ingeniería Agroindustrial, Universidad Nacional de Moquegua, Prolongación Calle Ancash s/n, Moquegua, CP 18001, Peru olimpiallcr@gmail.com

[4] Departament de Biologia, Sanitat i Medi Ambient, Facultat de Farmàcia i Ciències de l'Alimentació, Universitat de Barcelona, Av. Joan XXIII 27-31, 08028 Barcelona, Spain, ruscheltallini@ub.edu, lauratorrascl@ub.edu

[5] Herbario del Sur de Bolivia, Instituto de Botánica y Ecología, Universidad Mayor Real y Pontificia de San Francisco Xavier de Chuquisaca, Calle Junín Esq. Estudiantes 692, Sucre, Bolivia hiberhuaylla@gmail.com

[6] Grupo Biología y Control de Enfermedades Infecciosas – BCEI, Universidad de Antioquia UdeA, Calle 70 No. 52-21, Medellín, Colombia; maría.mejia3@udea.edu.co, omar.triana@udea.edu.co

[7] Grupo de Investigación en Sustancias Bioactivas GISB, Facultad de Ciencias Farmacéuticas y Alimentarias, Universidad de Antioquia UdeA, Calle 70 No. 52-21, Medellín, Colombia; edison.osorio@udea.edu.co

* Correspondence: G.E. Feresin gferesin@unsj.edu.ar

Table S1. UPLC-ESI-MS/MS data for *Eustephia hugoei* (EB5) alkaloid extract

Alkaloid	Type	[M+H]+	Base peak	RT	Area	%TIC
Candimine	Homolycorine	346	221	5,39	6003082,5	12,15
NI	Homolycorine	332	207	5,96	1478101,3	2,99
NI	Homolycorine	318	318	6,41	452884,0	0,92
NI	Homolycorine	362	268	6,51	2053415,6	4,16
NI - Nerinine?	Homolycorine	348	237	6,64	775230,3	1,57
NI	Lycorine	302	162	6,96	766280,8	1,55
NI	NI	284	266	7,67	926519,1	1,88
Homolycorine	Homolycorine	316	316	8,00	1006333,6	2,04
NI	Homolycorine	346	237	8,21	12650139,0	25,60
NI	Lycorine	252	237	9,22	3582804,8	7,25
NI	Homolycorine	362	297	10,14	4585366,5	9,28
NI	Lycorine	266	250	11,48	10024448,0	20,29
NI - Hydroxyalbomaculine?	Homolycorine	362	221	15,48	5100860,0	10,32
**Total**					**49405465,38**	**100,00**

Table S2. UPLC-ESI-MS/MS data for *Eustephia darwinii* (EB4) alkaloid extract

Alkaloid	Type	[M+H]+	Base peak	RT	Area	%TIC
NI	Homolycorine	360	221	4,46	706978,69	2,21
Hippeastrine	Homolycorine	316	316	4,66	1738954,38	5,44
Candimine	Homolycorine	346	221	5,04	14249077,00	44,58
NI	Homolycorine	332	207	5,74	492411,78	1,54
NI	NI	362	85	6,37	421958,69	1,32
NI	NI	360	122	7,83	755471,56	2,36
NI	NI	346	337	8,13	10251682,00	32,07
NI	Homolycorine	362	332	9,92	994170,56	3,11
NI - Hydroxyalbomaculine?	Homolycorine	362	221	15,00	2352036,25	7,36
**Total**					**31962740,91**	**100,00**

Table S3. UPLC-ESI-MS/MS data for *Eustephia* coccinea (EB3) (Tinta - Cusco) alkaloid extract

Alkaloid	Type	[M+H]+	Base peak	RT	Area	%TIC
Pseudolycorine	Lycorine	290	147	1,68	354248,56	1,17
NI	NI	334	109	2,56	1442718,88	4,75
NI	Haemanthamine	288	288	3,08	460155,91	1,52
NI	Haemanthamine	288	185	3,98	567332,13	1,87
NI	Haemanthamine	302	222	5,01	118367,81	0,39
NI	NI	332	134	5,35	10683214,00	35,18
Galanthine	Lycorine	318	162	6,55	455111,56	1,50
NI	NI	318	192	6,74	347706,78	1,14
Haemanthidine	Haemanthamine	318	199	7,12	1375606,50	4,53
NI	NI	348	228	7,35	1695721,13	5,58
Haemanthamine	Haemanthamine	302	196	7,62	11555786,00	38,05
NI	NI	318	225	8,22	657535,44	2,17
NI	NI	268	253	8,25	657535,44	2,17
**Total**					**30371040,13**	**100,00**

Table S4. UPLC-ESI-MS/MS data for *Eustephia* coccinea (EB2) (Pisac - Cusco) alkaloid extract

Alkaloid	Type	$[M+H]^+$	Base peak	RT	Area	%TIC
NI	Haemanthamine	302	222	5,42	620209,38	4,52
NI	NI	318	318	6,33	178650,27	1,30
NI	NI	320	268	6,46	380926,31	2,77
NI	NI	304	286	6,64	584697,56	4,26
NI	NI	304	286	7,11	406775,50	2,96
NI	NI	316	211	7,31	351661,84	2,56
Haemanthamine	Haemanthamine	302	196	7,68	1045572,63	7,61
NI	Haemanthamine	318	318	8,25	699422,13	5,09
NI	NI	252	237	9,11	1818711,00	13,24
NI	NI	252	237	9,26	733155,00	5,34
NI	NI	346	268	10,97	531977,75	3,87
Assoanine	Lycorine	266	250	11,26	6380358,50	46,46
**Total**					**13732117,86**	**100,00**

Table S5. UPLC-ESI-MS/MS data for *Eustephia* coccinea (EB1) (Taray - Cusco) alkaloid extract

Alkaloid	Type	$[M+H]^+$	Base peak	RT	Area	%TIC
NI	Lycorine	290	147	1,39	125239,58	0,44
Lycorine	Lycorine	288	147	2,31	83699,66	0,29
NI	Haemanthamine	302	213	2,58	361399,09	1,27
NI	Haemanthamine	288	196	3,09	429950,47	1,51
Galanthamine	Galanthamine	288	213	3,50	987835,38	3,46
Hippeastrine	Homolycorine	316	96	4,65	825690,88	2,89
NI	Galanthamine	304	213	4,91	671976,81	2,36
Candimine	Homolycorine	346	221	5,07	7980451,50	27,97
NI	Haemanthamine	302	-	5,35	830813,00	2,91
NI	Homolycorine	332	207	5,72	1036079,44	3,63
NI (overlapped)	NI	332/362	-	6,40	831388,81	2,91
Galanthine	Lycorine	318	162	6,56	3372373,75	11,82
NI	Lycorine	139	332	7,07	254839,75	0,89
NI	Haemanthamine	316	211	7,30	2429805,25	8,52
Haemanthamine	Haemanthamine	302	196	7,65	3215960,00	11,27
NI	NI	268	253	8,11	272433,19	0,95
NI	NI	318	225	8,25	17422,88	0,06
NI	NI	282	266	10,86	933356,19	3,27
NI	Homolycorine	362	221	14,83	359696,53	1,26
NI	NI	296	280	16,80	3511045,00	12,31
**Total**					**28531457,15**	**100,00**

Table S6. GC-MS spectra of the identified alkaloids in *Eustephia* species from Peru.

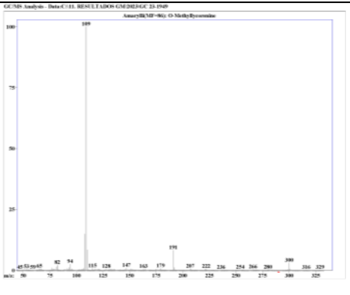	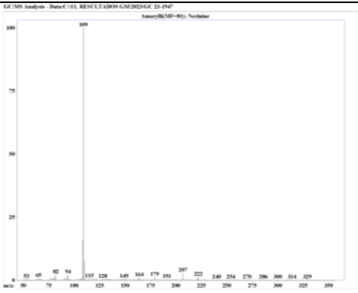	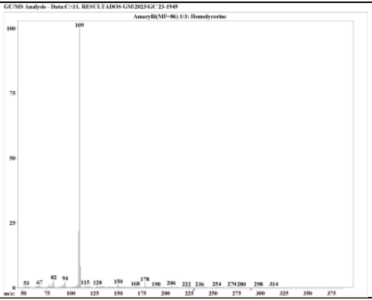
1. O-Methyllycorenine	2. Nerinine	3. Homolycorine
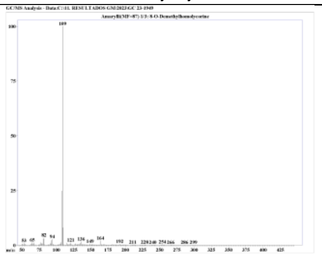	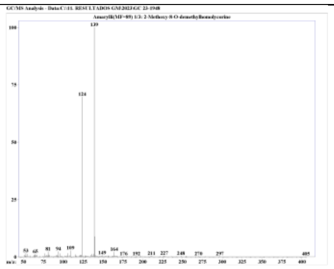	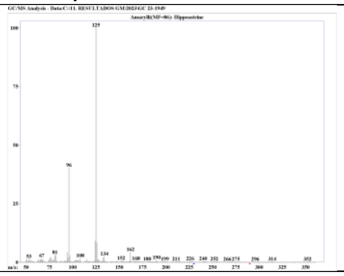
4. 8-O-Demethylhomolycorine	5. 2-Methoxy-8-O-Demethylhomolycorine	6. Hippeastrine
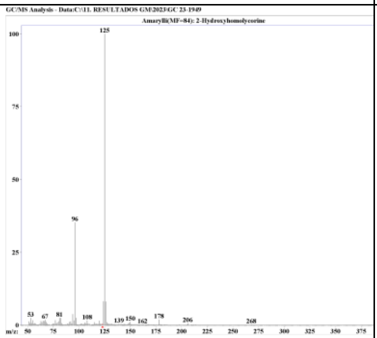	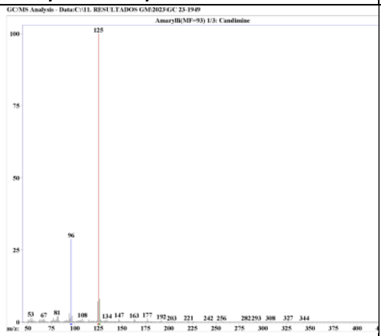	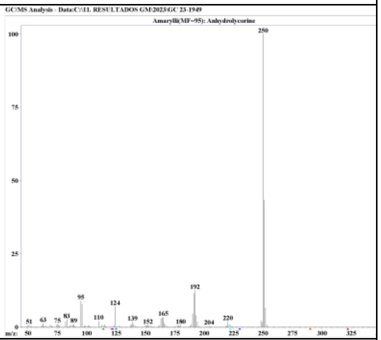
7. 2-Hydroxyhomolycorine	8. Candimine	9. Anhydrolycorine
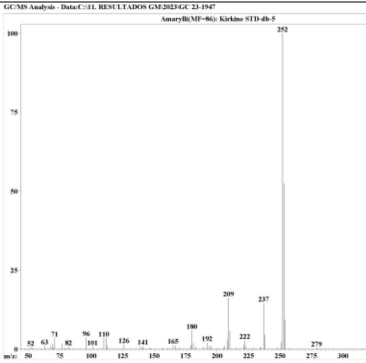	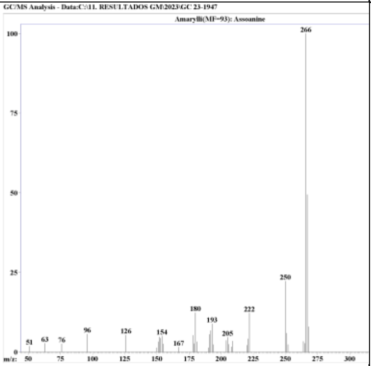	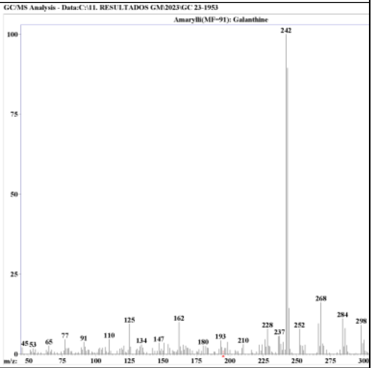
10. Kirkine	11. Assoanine	12. Galanthine

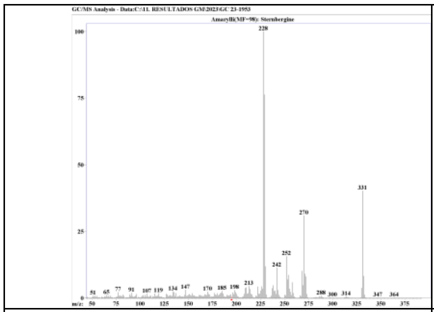	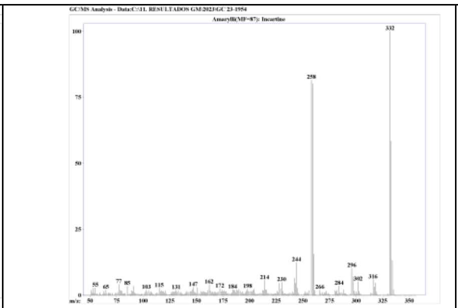	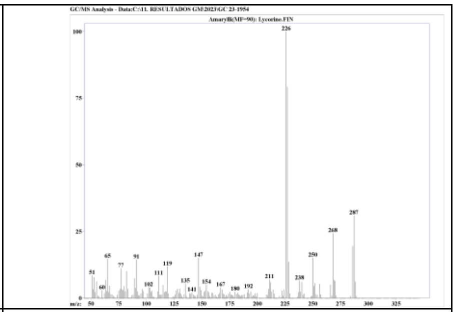
13. Sternbergine	14. Incartine	15. Lycorine
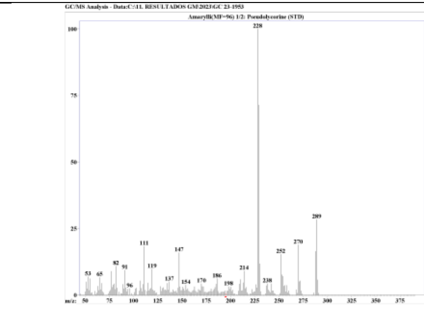	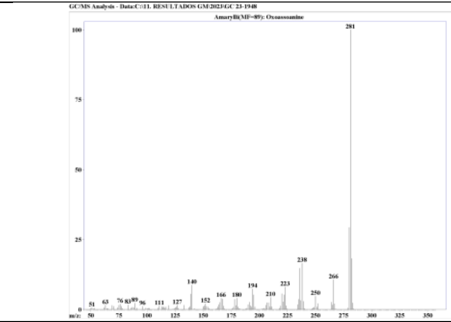	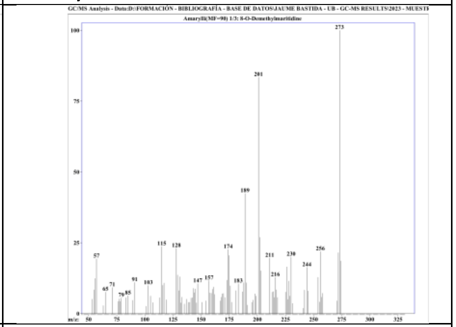
16. Pseudolycorine	17. Oxoassoanine	18. 8-O-demethylmaritidine
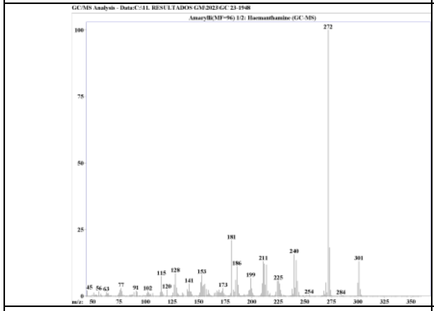	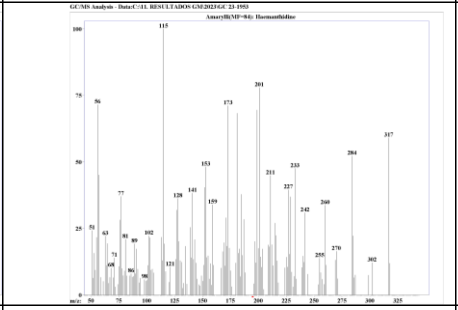	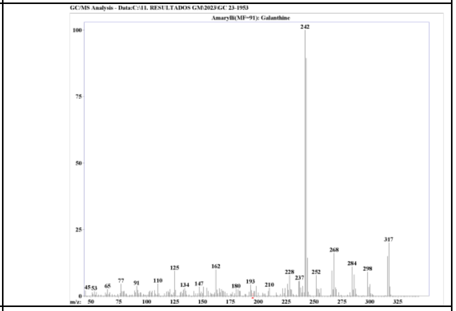
19. Haemanthamine	20. Haemanthidine	21. Galanthamine
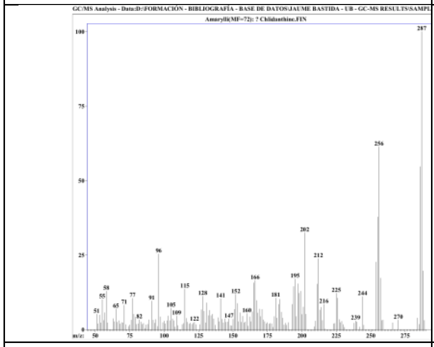	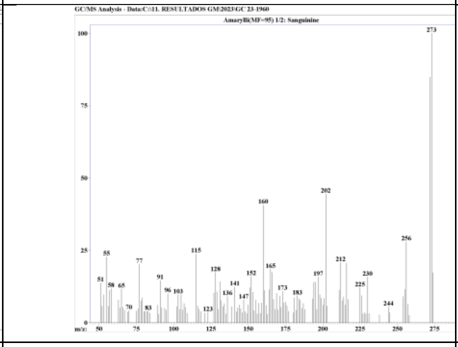	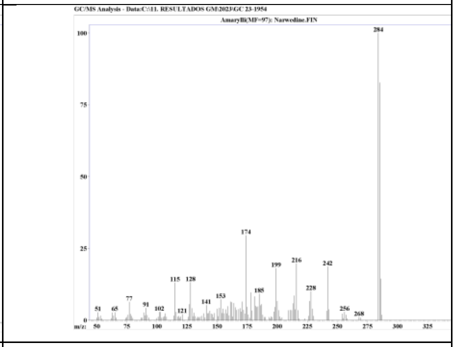
22. Chlidanthine	23. Sanguinine	24. Narwedine
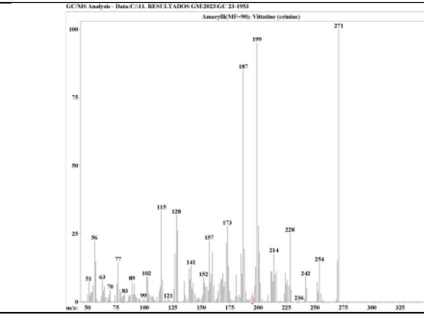	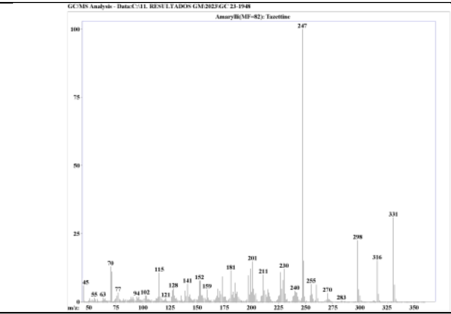	
25.Vittatine/crinine	26. Tazettine	
